# Lymphangioma of the Lower Lip—A Diagnostic Dilemma: Report of a Rare Case with a Brief Literature Review

**DOI:** 10.1155/2022/7890338

**Published:** 2022-06-02

**Authors:** Shamimul Hasan, Syed Ansar Ahmad, Mandeep Kaur, Rajat Panigrahi, Swagatika Panda

**Affiliations:** ^1^Department of Oral Medicine and Radiology, Faculty of Dentistry, Jamia Millia Islamia, New Delhi, India; ^2^Department of Oral and Maxillofacial Surgery, Faculty of Dentistry, Jamia Millia Islamia, New Delhi, India; ^3^Department of Oral Medicine and Radiology, Institute of Dental Sciences, Siksha ‘O' Anusandhan University, Bhubaneswar, Odisha, India; ^4^Department of Oral and Maxillofacial Pathology, Institute of Dental Sciences, Siksha ‘O' Anusandhan University, Bhubaneswar, Odisha, India

## Abstract

Hamartomas are tumor-like deformities typified by cellular propagation indigenous to the original site, although they display growth arrest without the possibility for further growth. Various hamartomatous oral lesions include hemangiomas, lymphangiomas, nevi, odontomas, Cherubism, etc. Lymphangiomas are benign, developmental hamartomatous entities typified by abnormal proliferation of lymphatic vessels. They are usually congenital, and more than 90% of cases occur by 2 years of age, with a rare occurrence in adults. They have a site affinity for the head and neck, and oral lesions are relatively uncommon. The dorsum of the tongue is the commonest oral site of predilection; however, the lip is a rare site of involvement. Hereby, we present an uncommon case of lymphangioma of the lower lip in a 45-year-old male patient, who reported to our hospital with an asymptomatic lower lip submucosal mass present for the last 3 years. Our case is unique as it occurred on the lower lip of a 45-year-old male. A detailed history and clinical evaluation, ultrasonography, and histopathology confirmed the diagnosis of lymphangioma.

## 1. Introduction

Hamartoma is described as a benign, unifocal/multifocal, developmental abnormality, encompassing a blend of cytologically normal mature cells and tissues native to the anatomic site, and exhibits a disordered architectural outline with a preponderance of one of the elements [1].

Lymphangioma refers to hamartomatous entities originating from abnormal lymphatics which cease to drain into other lymphatics or veins, thus causing lymph stagnation resulting in the development of large cyst-like dilated lymphatics [2, 3]. Lymphangiomas are typified by aberrant propagation of lymphatic vessels and are considered nonneoplastic developmental anomalies rather than actual tumors [4–6].

Owing to the intimate proximity to the primitive jugular lymphatics, most cases occur in the head and neck region (75%), followed by the clavicle and axillary regions (25%) [7–11]. The oral cavity is an infrequently involved site, with most occurrences seen in the anterior 2/3^rd^ of the dorsal aspect of the tongue. However, occasional cases may involve the palate, cheek, gingiva, lips, and alveolar ridges [4, 7, 9–13]. Infants and children below the age of 2 years are mostly affected, with uncommon occurrences in adults [3, 6, 12, 14].

Hereby, we present an unusual case of lymphangioma of the lower lip in a 45-year-old male patient. Our case is unique in a few aspects as it manifested age and site variation.

## 2. Case Description

**Figure 1:**
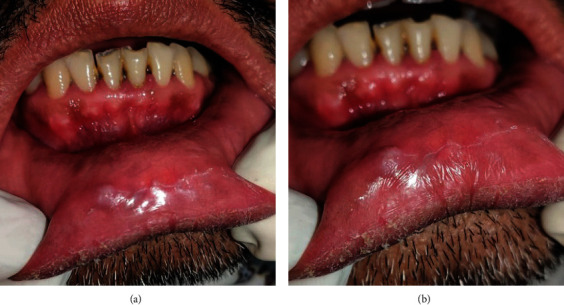
(a, b) Solitary, well-circumscribed submucosal mass in the lower lip, with an adjacent bluish-white swelling depicting a mucocele.

A 45-year-old male patient with no known comorbidities reported to our Outpatient Department with a complaint of a mass in the lower lip region for the past 3 years. The initially smaller mass has shown gradual progression, although the lesion has been static for the past 3-4 months. There was a negative history of similar lesions in any other part of the body. However, there was an occasional factitial habit of lip biting. The extraoral examination was unremarkable. On intraoral examination, a solitary, well-delineated nodular mass was noticeable in the submucosa of the lower lip, 2 × 1.5 cm in diameter, extending anteriorly 3 cm away from the mucocutaneous junction, posteriorly blending and obliterating the vestibule, and mesiodistally extending from right canine to the left lateral incisor region. The overlying mucosa was of the same color as the adjacent mucosa, smooth-surfaced with few interspersed areas exhibiting a granular appearance. Palpatory findings were suggestive of a nontender, nonfluctuant mass, soft to firm in consistency, nonpulsatile, and not fixed to the adjacent tissues. Multiple grainy nodules were also felt on palpation. The lesion did not blanch on pressure, and there was no sensory deficit in the lip region. A solitary, sessile, bluish-white, soft, and nontender swelling, 3.5 × 2.5 mm in diameter, was also seen on the right aspect of the vermilion border with mild hyperkeratinization, features in sync with the factitial lip biting. The patient's oral hygiene was fair and class II was seen with 31 (Figures 1(a) and 1(b)). The tooth fracture occurred around 20 days back while biting on a peanut, and the tooth was nondiscolored and asymptomatic.

## 3. Differential Diagnosis

Considering the anamnesis and clinical appearance of a localized, slow-progressing, smooth-surfaced, well-defined, asymptomatic, freely movable mass located within the lower lip submucosa, the lesion was considered of benign origin. Entities of minor salivary gland origin (mucocele, salivary duct cysts, and benign and malignant minor salivary gland tumors) and infectious origin (odontogenic/nonodontogenic) and mesenchymal tumors of adipose tissue (lipoma), vascular entities (hemangioma and lymphangioma), smooth muscle lesions (leiomyoma), or neural derivatives (traumatic neuroma, schwannoma, and neurofibroma) were given a place in the list of differential diagnosis.

The lower lip is extremely vulnerable to factitial injury and has a propensity for minor salivary gland tissues; thus, mucocele or salivary duct cysts account for the majority of swellings/lumps in this area. In the present case, the probability of the mass being a mucocele may be negated by its large size (mucoceles larger than 1.5 cm in diameter are extremely infrequent) [10], age of the patient (mucoceles are more likely to occur in children and young adults) [10, 15], chronic duration of 3 years, soft-firm consistency, and covered with normal overlying mucosa. However, a solitary, bluish-white colored, soft, and nontender swelling in the right aspect of the vermillion border with an associated occasional lip biting may be clinically diagnosed as a mucocele.

The possibility of salivary duct cysts is highly unlikely as they characteristically appear as bluish, firm nodular swellings on the floor of the mouth and rarely on the lips [16].

Benign minor salivary gland tumors (primarily pleomorphic adenomas and canalicular adenomas) primarily involve the upper lip, with infrequent occurrence on the lower lip. Moreover, pleomorphic adenoma is usually seen in younger individuals (<40 years), and canalicular adenoma has an elderly female predilection (>60 years) [17]. These features contrast with the present case.

The diagnosis of a malignant minor salivary gland tumor (adenoid cystic carcinoma and acinic cell carcinoma) may be ruled out based on the location, well-circumscribed mass, chronic duration, and lack of fixation [18].

Soft tissue infections of the lip (odontogenic/nonodontogenic) may be negated due to the asymptomatic and chronic duration of the lesion with normal dentition. Soft tissue lip abscess is generally ill-defined and fluctuant and exhibits inflammatory signs (pain and erythema) [19].

Lipoma is a nonneoplastic adipose tissue tumor, with an uncommon (1-4%) occurrence in the oral cavity. Lipomas characteristically manifest as a slow-growing, yellow-colored, soft, asymptomatic submucosal mass, with a typical “slip sign,” occurring mostly on the tongue and cheek region [20].

Hemangioma diagnosis may be negated taking into account the patient's age and gender (hemangiomas occur exclusively in infancy and young adults, especially in girls), chronicity of the lesion, noncompressibility, lack of a peculiarly bright red lesion, and a negative diascopy test [16, 19].

Lymphangioma, a benign hamartomatous lesion of the lymphatic system, primarily affects infants and children and involves the tongue, although few lip cases have also been documented [3]. The lip lesions frequently exhibit an asymmetric, asymptomatic, firm, and nodular pattern [19].

Leiomyoma, a benign smooth muscle tumor, infrequently occurs in the oral cavity due to the absence of smooth muscle in the region [21]. The lesion mostly affects individuals in the 3^rd^ decade and manifests as a slow-progressing, asymptomatic mass.

Reactive and neoplastic neural lesions (traumatic neuroma, neurofibroma, and schwannoma) may also be considered in the present case. The oral traumatic neuroma typically manifests as a nodular lesion, in close proximity to the mental foramen, tongue, or lips. However, the lesions are painful on palpation [22].

Schwannomas (neurilemmomas) are benign neural tumors and manifest as discrete, firm, smooth-surfaced nodular lesions of the same color as the adjacent mucosa. They have a site affinity for the head and neck region, although lip involvement is an extremely rare occurrence [17, 23].

Neurofibroma (NF) is a nerve sheath neoplasm, manifesting as a localized lesion or as a component of the diffuse neurofibromatosis syndrome. However, solitary NF is an extremely unusual occurrence in the oral cavity (6% occurrence) and infrequently affects the lower lip [23].

To summarize, our clinical impression was suggestive of a benign minor salivary gland lesion.

## 4. Investigations and Treatment Plan

With these differentials in mind, the patient was subjected to further investigations. Negative results were obtained on diascopy with a glass slide and fine-needle aspiration (FNA) of the mass that revealed blood-tinged clear fluid resembling lymphatic fluid. The minimally invasive procedure of FNA not only aids in the initial tissue-based diagnosis of salivary gland lesions but also aids in sorting and contemplating the treatment strategy for the patient [24].

**Figure 2:**
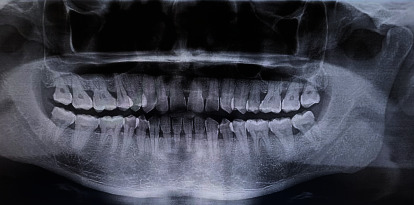
OPG demonstrating an ill-defined periapical pathology with #31.

Hematologic evaluation including complete blood count, serum calcium, phosphorus, and parathormone levels was within normal limits. A low platelet count (thrombocytopenia) may indicate a risk of bleeding prior to surgical interventions, with a plethora of bleeding symptoms such as bruising, nosebleeds, and, rarely, grievous or fatal bleeding [25]. Orthopantomogram (OPG) was unremarkable except for the presence of an ill-defined periapical pathology i.r.t #31 (Figure 2).

**Figure 3:**
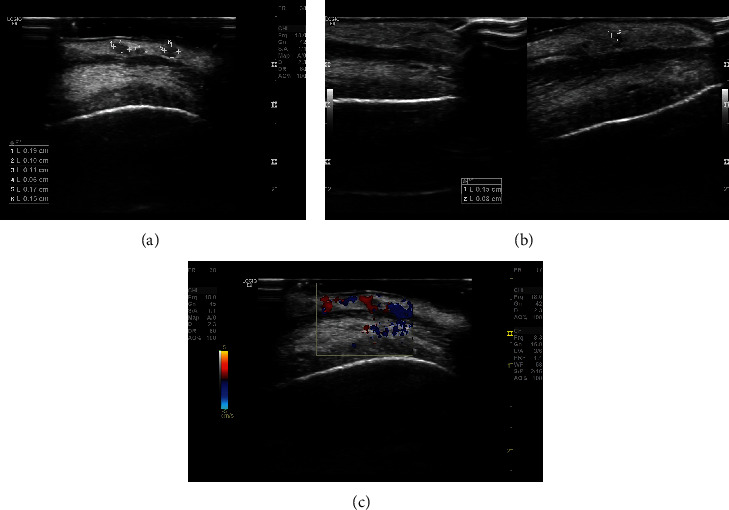
(a, b) Adjacent tiny lesions on the right and left sides of the lip. (c) The color Doppler was suggestive of an avascular anechoic lesion.

Ultrasound was done using a linear probe with special 2D enhancement features including compound and speckle reduction imaging (SRI). The study revealed tiny anechoic cystic lesions with internal septa, measuring approximately 1.9 × 1.0 mm (clinically visible lesion) with adjacent tiny lesions measuring 1.1 × 0.6 mm and 1.7 × 1.5 mm (Figure 3(a)). Another similar morphological lesion was apparent on the left side measuring 1.5 × 0.8 mm (Figure 3(b)). The color Doppler was suggestive of an avascular anechoic lesion (Figure 3(c)). USG serves as a noninvasive aid for the diagnosis of soft tissue lesions, delineation of cystic from solid lesions, and benign from malignant masses. The color Doppler USG may be employed to ascertain the presence or absence of vascular flow in normal tissues and in diseased states [26].

Magnetic resonance imaging (MRI) of the lower lip was advised, but the patient denied the investigation due to financial constraints.

**Figure 4:**
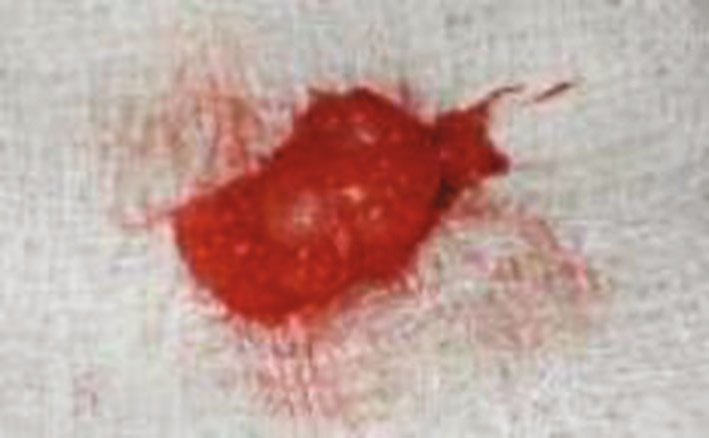
Gross excised specimen was suggestive of a firm nodular mass.

After taking written consent from the patient, an excisional biopsy was done under local anesthesia. The lesion was enucleated via a blunt horizontal incision of the mucosa with no. 15 blades, taking utmost care not to injure the vital structures. Fine-dissecting scissors and artery forceps were used to excise the lesion. 3-0 vicryl was used to suture the muscle bed, and the lip was approximated and sutured with silk. Grossly, the excised specimen was a nodular mass, firm in consistency, and measured 3 × 2 cm (Figure 4). The enucleated specimen was submitted for histopathological examination.

**Figure 5:**
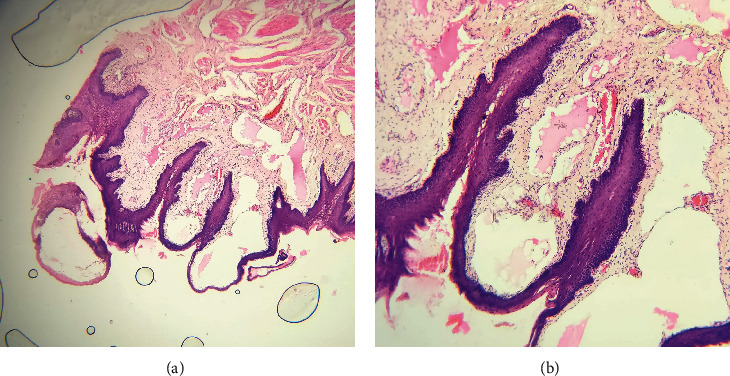
(a) The photomicrograph (10x) reveals the atrophic epithelium beneath which there are numerous dilated lymphatic channels lined by endothelial cells. (b) The photomicrograph (400x) shows large lymphatic channels below the stratified squamous epithelium.

Histological evaluation of the hematoxylin and eosin-stained sections (10x) reveals an atrophic epithelium beneath which there are numerous dilated lymphatic channels lined by endothelial cells within the papillary and reticular layers of the lamina propria. The large dilated lymphatics just are filled with lymph and infiltrated with inflammatory cell infiltrates, predominantly lymphocytes, neutrophils, and plasma cells. The histopathological features were in coherence with a diagnosis of lymphangioma (Figure 5). However, the patient was lost for further follow-up.

## 5. Discussion

There exists a diverse pattern in defining lymphangiomas; however, the nonneoplastic proliferation of lymphatic channels delineating a lymphatic hamartoma represents the classical description of lymphangiomas [27].

Lymphangiomas emanate from the sequestration of primordial lymphatic cells. The lymphatic channels persistently accumulate lymph; however, they fail to join with the larger vessels, causing lymphatic obstruction, thus imparting a cystic appearance [7]. They are rare entities with an incidence of about 1 : 6,000 to 1 : 16,000 live births and are attributed to about 6% of benign, 11% of maxillofacial tumors, and 9% of soft tissue tumors in the pediatric age group [3, 27, 28].

Embryologically, lymphangiomas are mostly congenital (50%); the majority of the lesions (90%) manifest by 2 years of age or in rare cases, which may be acquired [3, 7, 9–12, 28–30]. The probable predisposing factors for acquired lymphangiomas include long-standing trauma, inflammation, and lymphatic blockage due to infection, radiotherapy, or surgery.

Congenital lymphangiomas can also occur in association with hemangiomas, certain chromosomal aberrations (Noonan syndrome, Turner syndrome, and Down syndrome), fetal hydrops, fetal alcohol syndrome, cardiac anomalies, and familial pterygium colli [12, 13, 31]. Lymphangioma in adults is an uncommon occurrence, with only a few documented cases [3, 7, 11, 14, 29–31].

Our patient was a 45-year-old male with an occasional factitial habit of lip biting. The repeated local trauma in the lower lip region could have triggered the proliferation of lymphatic vessels.

The close anatomical proximity to the primordial jugular lymphatic sac probably justifies the lymphangioma site predilection for the head and neck region (90% of cases seen in the head and neck region) [6–11]. The oral cavity is rarely the site of occurrence, and the commonest affected oral site is the anterior 2/3^rd^ of the dorsum of the tongue (often resulting in macroglossia) [9, 12, 13]. Other affected oral sites in decreasing order are the palate, buccal mucosa, gingiva, floor of the mouth, and lips [4, 7, 9–14, 31].

The symptoms of adult-onset lymphangioma are atypical and vary considerably, thus, posing a diagnostic dilemma [32].

Lymphangiomas of the lower lip are unusual and account for <1% of the lip biopsies. Lip lesions clinically mimic mucocele, thus causing a diagnostic difficulty [10], as seen in the present case. Lip involvement and the associated anomalies are described as macrocheilia [12, 31].

An extensive literature search was carried out on the Google Scholar and PubMed search engines using the following keywords: lymphangioma, lymphatic malformation, oral cavity, lip, and lower lip. Case reports published in the English language up to December 2021 were thoroughly searched. This extensive bibliographic research finally resulted in 16 case reports, after excluding studies that were duplicates, not case reports, and those which were not in the English language.

The parameters extracted from these cases are summarized in Table 1, and they concerned the following: year of occurrence, age and gender, age at the time of initial presentation, location, size, chief complaint, and treatment. Out of the 16 reported cases, 7 cases were seen in males, in the age range of 14 months-69 years, size range from 0.3 cm to 4 cm, and mostly manifesting as swelling/enlargement. Most of the cases were treated by surgical excision, with few cases exhibiting recurrences.

The reported cases of lip lymphangiomas are summarized in Table 1.

The lesion extent directs the clinical appearance of lymphangiomas [3, 4, 11, 14]. They frequently manifest as a long-standing, asymptomatic, progressively expanding soft tissue mass that has been reasonably inactive, with intermittent episodes of exacerbation and remission, eventually remaining as a residual mass [3, 12, 31]. Superficial lymphangiomas typically manifest as pebbly, vesicle-like lesions, with the characteristic “frog-egg” or “tapioca pudding” presentation, although occasionally, the vesicles may appear purplish/bluish-red owing to the secondary hemorrhage into the lymphatic system [3, 10, 13, 14, 27]. However, a soft, diffused nodular submucosal mass with a similar texture and color as the contiguous mucosa depicts the classical description of deep-seated lymphangiomas [3, 12–14].

Our case presented as a solitary, slow-growing, well-circumscribed, asymptomatic, soft-firm, nonblanching, nodular submucosal mass in the lower lip region with normal overlying mucosa, although a few interspersed areas exhibited a granular appearance. A solitary, sessile, bluish-white, soft, and nontender swelling, 3.5x 2.5 mm in diameter, was also seen (in proximity to the mass) on the right aspect of the lip vermilion border with mild keratinization.

Several detrimental effects such as breathing and speech difficulty, pain, tongue protrusion, or jaw anomalies may be seen with deep lesions owing to the impingement on the contiguous anatomical structures [3, 13, 27].

Secondary infection may predispose to Ludwig's angina. The postsurgical sequalae may include bleeding, seroma formation, repeated episodes of cellulitis, and leakage of lymphatic fluid [33].

Several hypotheses have been recommended for the etiopathogenesis of lymphangiomas. The first hypotheses propose that congenital lymphangiomas result from sequestration of primordial lymphatics; however, they fail to connect to the main lymphatic vessels or veins [34]. The second hypothesis states that the failure of the lymphatic vessels to drain into the veins causes lymph accumulation and leads to the formation of large cyst-like dilated lymphatics [27]. According to the third hypothesis, lymphangiomas may develop through aberrant budding of the lymphatic vessels, thus establishing new anomalous divisions. The fourth hypotheses suggest that long-standing inflammation may evoke lesion development by triggering the proliferation of lymphatic channels [29]. The most recent hypotheses document an increased expression of vascular endothelial growth factor-C (VEGF-C) and vascular endothelial growth factor R3 [35].

Histopathologically, lymphangiomas consist of dilated lymphatic vessels with one or two endothelial layers, with or without an adventitial layer. Based on the location and contiguous structures, these dilated lymphatic vessels are of variable sizes, thus forming the basis of classification. Cavernous lymphangiomas commonly occur in regions with a rich supply of denser connective tissue and skeletal muscle, causing confined lesions, e.g., oral cavity (dorsal aspect of the tongue, buccal mucosa, and floor of the mouth). This is contrary to cystic hygromas, which develop in loose adipose tissue sites resulting in a diffuse spread and large multicystic lesions [28, 34].

The microscopic findings in our case were incoherent with the published literature.

Most lymphangiomas are diagnosed clinically based on the characteristic manifestations. However, excision and histological assessment, together with diagnostic radiographic aids facilitate the confirmatory diagnosis of lymphangiomas with bizarre clinical characteristics [7, 34].

Ultrasound (USG) is an essential diagnostic aid in assessing the site, shape, size, and extent of lesions. Ultrasound generally exhibits multicystic lesions with internal septations, and the color-flow Doppler depicts the avascular nature of lymphangiomas, thus, not only distinguishing vascular malformations from hemangiomas but also identifying the various vascular patterns of such entities.

In our case, the USG findings were suggestive of multiple anechoic cystic lesions with internal septa on the right and left sides of the lower lip. The color Doppler was suggestive of an avascular anechoic lesion.

However, computed tomography (CT) and magnetic resonance imaging (MRI) are superior to ultrasound in describing the relationship with contiguous anatomical structures and demarcating the lesion extent [27, 29].

Magnetic resonance imaging (MRI) of the lower lip was advised, but the patient denied the investigation due to financial constraints.

Mostly, lymphangiomas are benign in origin; however, they may require surgical treatment due to their infiltrative pattern causing larger lesions to encroach the adjoining vital structures, resulting in cosmetic, functional, and life-threatening sequelae [3, 7].

The treatment strategies should focus on restoring the functions, averting any potential complications (secondary infections/internal hemorrhage), and attaining adequate cosmetic satisfaction [36]. A wide array of proposed management strategies for lymphangiomas are surgical excision, electrocautery, cryotherapy, radiotherapy, intralesional steroids, sclerosing agents (OK432), embolization and ligation, laser treatment with Nd:YAG and carbon dioxide, and radiofrequency tissue ablation technique. However, surgical excision is considered the gold standard of treatment [3, 11–14, 27]. Adult lymphangiomas are mostly encapsulated, thus facilitating the surgical excision [12, 27, 28, 30].

Sclerotherapy with 25% dextrose, hypertonic saline, bleomycin, and picibanil (OK-432) is considered deemed for recurrent, unresectable/surgically demanding lesions [37]. Sclerosing agents have shown superior efficacy in the management of macrocystic lymphangiomas, as compared to the microcystic lyphangiomas [38]. Senthilnathan et al. treated a soft tissue lymphangioma by ultrasonographic-guided bleomycin sclerotherapy. Intralesional bleomycin not only damages the endothelial lining of the lymphangioma but also stimulates the diffusion of the chemical into the cystic spaces, thus, causing thrombosis and fibrosis around the vascular spaces [39].

Recently published reports have also documented the successful management of tongue lymphangiomas by nonablative long-pulsed Nd:Yag lasers and constitutional homoeopathic medicine Tuberculinum, respectively [40, 41].

Surgical extirpation has an accompanying high recurrence rate (10-39%), primarily due to the deeper permeation of the lesion or incomplete surgical removal. Preoperative intralesional administration of sclerosing agents may prove beneficial in such cases [30].

In our case, surgical enucleation of the mass was done using a blunt horizontal incision under local anesthesia.

## 6. Conclusion

Lymphangiomas are common entities in the pediatric age group and are rarely encountered in adulthood. They may be frequently misdiagnosed owing to the bizarre clinic radiographic manifestations, and histopathologic evaluation provides the confirmatory diagnosis. Lymphangiomas uncommonly involve the oral cavity, with a rare occurrence in the lower lip. Lymphangiomas should always be given a place in the differential diagnosis of a localized submucosal mass of the lower lip.

## Figures and Tables

**Table 1:** 

S. No.	Author and year	Age and gender	Age at the initial presentation	Site	Size (cm)	Chief complaint	Treatment	Recurrence
1.	Nagata (1984)	27/M	5	Right buccal mucosa-lower lip	—	Mass lesion	Excision	Yes
2.	Fukuda M et al. (1989)	14 months/M	1 month	Right lower lip and buccal mucosa	Thumb-sized tumor	Swelling	Excised several times, cryosurgery treatment many times, vaporized by laser irradiation	Repeated recurrences
3.	Balakrishnan (1991)	4/M	1 week	Neck-submandibular region-bilateral buccal mucosa-right parotid region-both lips-tongue	—	Swelling-difficulty with breathing	Laser therapy	Yes
4.	Park (2002)	69/F	39	Mandible-lower lip	4 × 2 × 1	Irritation-discoloration	—	—
5.	Ertugul et al. (2008)	23/M	22.5 months	Lower lip	0.6 × 0.9	Cosmetic concern	Excisional biopsy	No
6.	Nagaoka (2008)	9/F	2	Lower lip	2.8 × 1.7 × 0.1	Swelling	Excision	—
7.	Jha (2012)	13/M	5	Lower lip	—	Enlargement	Excision	—
8.	Bindu (2013)	13/M	13	Lower lip	1.5 × 1	Enlargement	Excision	—
9.	de Carvalho (2015)	6/M	—	Lower lip	0.3	Mass	Excision	—
10.	de Carvalho (2015)	4/F	—	Lower lip	1	Mass-difficulty in eating and speaking	Excision	—
11.	Fernandes (2018)	68/F	62	Lower lip	—	Mass	Follow-up	No
12.	Matharu (2019)	4/F	Birth	Lower lip	0.55 × 0.17 × 0.7	Mass	Cryotherapy	No
13.	Flores (2020)	6/F	6	Lower lip	0.6 × 0.5 × 0.4	Asymptomatic nodules	Excision	No
14.	Santos et al. (2020)	56/F	48	Lower lip	—	White painless vesiculobullous lesion	Excision	—
15.	Kurude et al. (2020)	10/M	9	Lower lip and buccal mucosa	2–3 mm papules	Asymptomatic lesions on the right buccal mucosa with swelling of the cheek and lips on the right side	Sclerotherapy	—
16.	Ehtisham (2021)	55/M	54	Lower lip	—	Painless growth	Excision	—
17.	Present case	45/M	42	Lower lip	2 × 1.5	Mass	Surgical excision	—
